# Research on the Dynamic Characteristics of Perfluoroalkoxy Alkane Springs

**DOI:** 10.3390/ma16155289

**Published:** 2023-07-27

**Authors:** Jing Ni, Yujie Feng, Zhi Cui, Lihua He, Jingbo Sun

**Affiliations:** School of Mechanical Engineering, Hangzhou Dianzi University, Hangzhou 310018, China; nj2000@hdu.edu.cn (J.N.); 221010004@hdu.edu.cn (Y.F.); jingbosun@hdu.edu.cn (J.S.)

**Keywords:** PFA spring, dynamic properties, frequency, dynamic stiffness, ductility coefficient

## Abstract

Semiconductor cleaning system ultra-clean flow control pumps are critical equipment in the semiconductor industry. Among them, the perfluoroalkoxy alkane (PFA) spring is a pivotal component to control the pump, and its dynamic performance is crucial to ensure the efficient operation of the system. However, the dynamic performance of the spring is often affected by the operating frequency. This paper studied the effect of different working frequencies on the dynamic property of the spring through compression-cycle experiments under uniaxial sinusoidal excitation. The force–displacement curves under different compression frequencies were fitted to obtain the dynamic stiffness of the PFA spring under different cyclic loading frequencies. The variation in the spring’s hysteresis coefficient was evaluated using the hysteresis curves of different cyclic loading conditions. After 2 million compression experiments, the changes in dynamic stiffness, hysteresis coefficient, and spring height were investigated. The obtained results revealed that, as the frequency increases, the dynamic stiffness of the spring increases. The hysteresis coefficient of the PFA spring is the largest at 10 Hz and the smallest at 6 Hz. Upon conducting 2 million compression tests, it was discovered that the dynamic stiffness experiences the greatest attenuation rate of 4.19% at a frequency of 8 Hz, whereas the hysteresis coefficient undergoes the largest attenuation of 42.1% at a frequency of 6 Hz. The results will help to improve the design and application level of PFA springs.

## 1. Introduction

The perfluoroalkoxy alkane (PFA) material is highly valued for its exceptional mechanical, chemical, and abrasion resistance [1,2]. As a result, it has become an indispensable material for flow control components in various industries, including chemical equipment manufacturing, aerospace equipment manufacturing, and medical equipment manufacturing. In ultra-pure pumps, the PFA spring is a crucial component responsible for controlling the closing of the check valve. The PFA spring operates under cyclic dynamic load working conditions, and its dynamic performance is a critical indicator of its service performance. A spring’s dynamic performance is an essential metric in evaluating the overall service performance of the spring. Additionally, it serves as a key index in ensuring the stable operation of the entire fluorine pump system. To enhance the service performance of PFA springs, it is imperative to investigate their dynamic performance when subjected to dynamic loads of various frequencies.

Spring stiffness is the most basic performance metric of the spring. As the stiffness increases, the check valve’s leakage rate decreases. However, excessive spring stiffness requires more kinetic energy to open the check valve, impairing its regular operation. Thus, it is crucial to determine an optimal stiffness for the check valve spring that balances the reduction in the leakage rate with the requirement of sufficient kinetic energy for proper valve opening. Jian et al. [3] conducted a study on the correlation between spring stiffness and valve stability. They discovered that an increase in spring stiffness resulted in an increase in the fluid damping coefficient in the valve, which in turn increased the stability of the valve. Azzam et al. [4] proposed a commonly used formula for calculating the stiffness of a helical spring. The static stiffness of the spring is related to the material and structure of the spring. Ke et al. [5] provided the static stiffness formula for a helical spring with different materials and different structures and provided the spring structure design method for different performance outcomes. This formula is widely used for metal springs. Some researchers have verified that the static stiffness calculation formula for metal springs can be applied to composite helical springs [6,7].

Generally, a spring’s stiffness is designed under the assumption of static conditions. A large amount of research has shown that, in addition to the material and structure of the spring itself affecting its stiffness, changes in working frequency also significantly impact its stiffness. Through uniaxial compression experiments on metal springs, Deng et al. [8] verified that the spring stiffness of metal springs at low frequencies differs significantly from their stiffness in static states, with the spring stiffness increasing dozens of times. Lee et al. [9] used a method of superimposing vibrational modes and assumed that the geometric shape of the helical spring is composed of continuous and uniform elements. Then, by combining the geometric characteristics of the helical spring with the dynamic equations, they effectively used traditional spring formulas to obtain the dynamic stiffness formula for the helical spring quickly. Cheng et al. [10] built a discrete spring model. They used the finite element method to study the relationship between the preload, the excitation amplitude, the material damping, and the dynamic stiffness of the spring. They found that the vertical dynamic stiffness of the helical spring showed an overall fluctuating upward trend. The dynamic stiffness will change abruptly at the resonant frequency of the spring. Gu et al. [11] proposed an analytical spring model with an arbitrary shape of the variable coil diameter and spring pitch, explaining and predicting the dynamic vibration response of the nonlinear honeycomb spring. The model has high accuracy in estimating the static and dynamic stiffness of the nonlinear spring. Yang et al. [12] modeled the helical spring using beam elements, considering the interaction between the spring coils and providing the stiffness of the helical spring under compression loads through simulation calculations. Liu et al. [13] and others measured a car-quality spring system’s vertical and lateral dynamic stiffness and provided the results of various constant preload forces in the 60–600 Hz frequency range. The dynamic stiffness model of the suspension component they built matched well with the actual one.

Working frequency changes significantly impact a spring’s service performance. Launay et al. [14] studied the performance of high-performance polyethylene fibers under cyclic conditions of a wide range of frequencies (0–500 Hz) and temperatures (50–250 degrees Celsius). They found that the material’s viscosity increased with increasing frequency. Tan [15] studied the viscoelastic response of PTFE materials and found that the three types of PTFE-based materials examined all exhibited an increase and then a decrease in loss modulus. The pure PTFE material had a peak loss modulus at 2.5 Hz, while the T05 and T99 materials had peak loss moduli at 25 Hz. Zheng et al. [16] conducted cyclic loading experiments on damper specimens and studied the correlation between the damper design parameters and the related performance at different frequencies, obtaining a curve plate damper structure with the characteristics of simple structure, precise mechanical performance, and good stability. Faughnan et al. [17] studied the correlation between the dynamic mechanical properties and the fatigue behavior of PTFE materials. Sedla’k et al. [18] used a numerical simulation to analyze the mechanical response of NiTi superelastic springs in detail and compared the results with experiments. Through analysis of the fatigue test of the simulated behavior, the relationship between fatigue resistance and periodic changes in martensite volume fraction caused by cyclic mechanical loads was found. The rebound performance of CHSs for composite materials was found to be opposite to the stiffness of springs that did not undergo fatigue failure. That is, the number of fatigue cycles increased with the decrease in spring stiffness. Chen [19] mentioned in the study of composite spiral springs (CHSs) that the CHS’s static and dynamic performance jointly determine whether they can be used on a large scale in the engineering field. The static performance of a CHS determines its operating load range, and the dynamic performance determines the service life and safety performance of the CHS under dynamic load environmental conditions. Lee [20] studied the change in the stiffness of a high-carbon steel spring under 15 Hz cyclic load at 190 °C. It was found that the stiffness of the spring decreased rapidly within 50 min, and the stiffness remained basically unchanged after 800,000 cyclic loads. Some researchers [21,22] have conducted fatigue tests on metal springs, studying the relationship between different spring sizes, materials, and cycles. It was found that the spring structure changes the most in the compression surface area, and the reason for the spring’s performance attenuation is explained from the perspective of the metal structure change. Some researchers [23,24] modeled the vehicle-mounted spring using the finite element method and used the Dirlik, Lalanne, and narrow-band methods to analyze the fatigue life of the spring in the frequency domain. In this way, the spring can be optimized for the spring’s working conditions or a suitable working environment can be chosen for the spring.

The literature review indicates that extensive research has been conducted on the theoretical stiffness of metal springs and on the changes in the performance of springs under dynamic loads. However, only a few studies have focused on PFA springs. There are very few studies on the dynamic performance of PFA springs. In this paper, a PFA spring working in the ultra-pure pump was designed. The uniaxial cyclic compression test of the PFA spring analyzed whether the spring’s performance met the requirements in a specific frequency range of 6–14 Hz. The spring’s dynamic stiffness, dissipation coefficient, and dynamic relaxation were used to evaluate the PFA spring’s dynamic performance. The changes in the performance of PFA springs with increasing cyclic excitations were also explored. The suitable frequency for PFA spring operation was obtained. Such research is of great significance for the design and application of PFA springs; it can help optimize their performance and reliability and improve their application effectiveness in fields such as semiconductor manufacturing equipment. At the same time, this also provides a specific reference value for researching other material springs.

## 2. Experiment

### 2.1. Workpiece Material

The PFA material selected in this experiment was purchased from DAIKIN (Osaka, Japan). The material trademark was AP-231SH. Its physical properties are summarized in Table 1.

### 2.2. Spring Designing

In the early stages of this research, we conducted a systematic comparison and analysis of the advantages and disadvantages of various types of springs. Compared with ordinary springs, rectangular cross-section springs are easier to manufacture and have better stability, which is more suitable for meeting the high precision and strength requirements of ultra-pure pumps. With the guidance of the manufacturer’s technical manual, we successfully designed a spiral spring with a rectangular section based on the valve size and operating conditions.

According to the actual performance requirements of the spring, the outer diameter of the spring was D_1 ≤ 28 mm, and the static stiffness of the spring was 95 ≤ k ≤ 160 N/m. The spring’s static stiffness is a key parameter for check valve sealing and flow control, which has an important influence on the working characteristics of the check valve.

The static stiffness formula of the rectangular spring is [26]

(1) $$K=\frac{4GI_{p}}{\pi D^{3}n}$$

where $I_{P}=k_{1}ab^{3}$ denotes the spring polar inertia distance. In this article, $k_{1}$ is 0.1869 [27]. *G* is the spring shear modulus.

(2) $$G=\frac{E}{2\left(1+u\right)}$$

where *E* denotes the bending modulus of the spring; *u* is the Poisson’s ratio.

The rectangular helical spring’s static stiffness can be obtained as

(3) $$K=\frac{4Ek_{1}ab^{3}}{\pi \left(1+u\right)D^{3}n}$$

**Figure 1:**
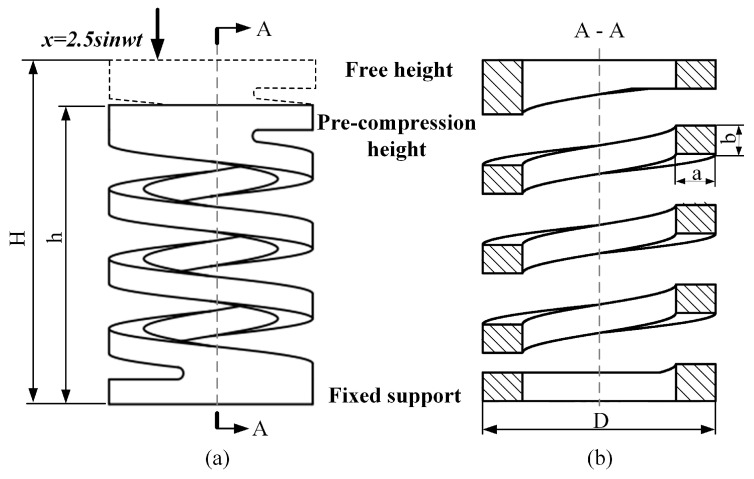
Spring structure diagram.

The specific structure of the spring is shown in Figure 1, and the structural parameters are shown in Table 2. In Figure 1a, H represents the free height of the spring, and h represents the height of the compressed spring. Figure 1b is the sectional view of the spring.

As shown in Table 3, the static stiffness of the spring was measured experimentally to be 120.85 N/mm. According to Formula (3), the calculated static stiffness of the spring is 123.29 N/mm. Compared to the experimental result, the error is 2.04%. This result showed that the static stiffness of the designed spring is within the design requirement range.

### 2.3. Test Device and Scheme

**Figure 2:**
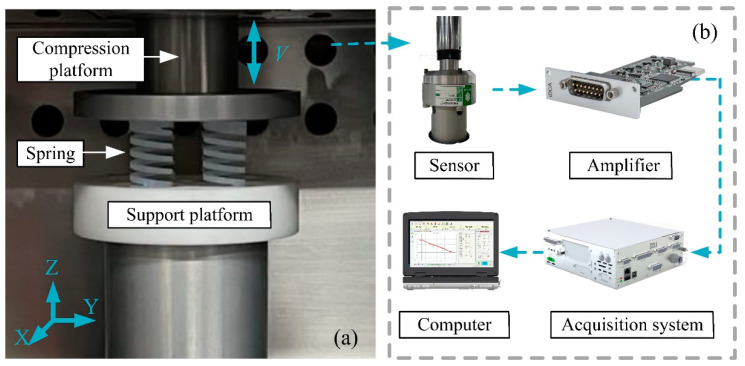
Experimental setup for: (**a**) compression system, (**b**) detail of compression.

**Figure 3:**
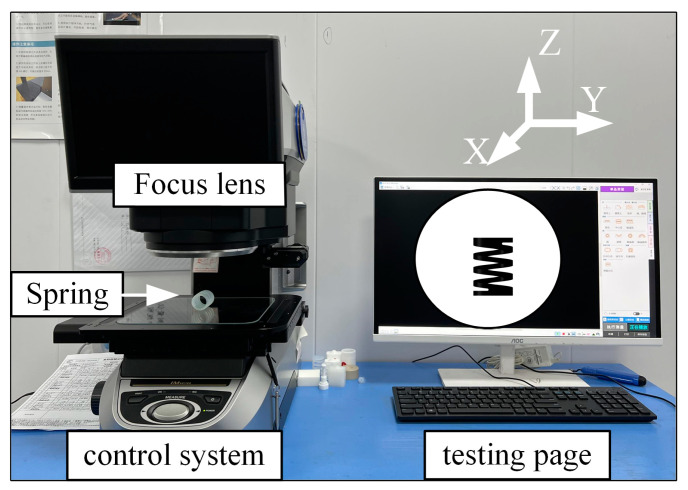
Spring size measurement system.

A uniaxial compression-cycle mechanics experiment was conducted on the spring to investigate the effects of the excitation frequency on stiffness, energy consumption levels, and dynamic relaxation. Experimental tests were conducted using the electronic spring fatigue test system, CTM-EF005 (Jinan, Shandong Province, China), as depicted in Figure 2a. The compression test was carried out in a thermostat (GW030) at 320 K. The compression rod was positioned at the upper part of the spring, while the support platform was at the lower part. A computer controls the displacement of sine waves of varying frequencies through a pressure bar. The experimental data were measured using the Celtron sensor (Malvern, PA, USA), as demonstrated in Figure 2b. The signals were transmitted to the charge amplifier (EDCi50), analyzed by the data acquisition system (IDCA), and finally displayed on the supporting software interface. Considering the spring shrinkage, as shown in Figure 3, the change in the free height of the PFA spring before and after the experiment was recorded using a KEYENCE IM-8020 (Osaka, Japan) image dimensional measuring instrument.

**Figure 4:**
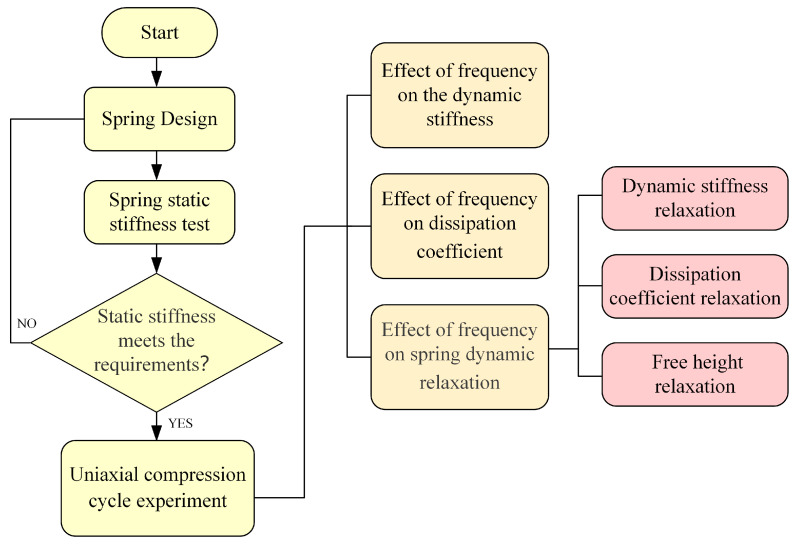
Flowchart of research.

The experimental scheme, as outlined in Table 4, involved the use of five sets of the same PFA spring. Before the experiment, the free height of the spring was measured at 38 mm. To apply the required pre-compression, the spring was compressed to a height of 35.5 mm, resulting in a pre-compression of 2.5 mm. To evaluate the dynamic characteristics of the PFA spring, a sinusoidal displacement excitation was applied to each of the five groups of springs. The cyclic frequencies used in the experiment were 6 Hz, 8 Hz, 10 Hz, 12 Hz, and 14 Hz, and the compression amplitude was set at 2.5 mm. During the experimental process, the changes in spring reaction force during the cyclic test and the final change in spring free height were recorded. The experimental steps are shown in Figure 4.

## 3. Results and Discussion

### 3.1. Dynamic Stiffness of PFA Spring

Stiffness refers to the ability of a structure to resist deformation. It is the most fundamental characteristic of a spring and is also a priority factor when selecting a spring. It is worth emphasizing that the PFA spring studied in this paper is the check valve spring of the pump. It works under repeated loading and unloading excitation conditions. Unlike the performance under static conditions of single compression, the stiffness of the PFA spring will change significantly with the change in excitation frequency. Therefore, it is necessary to use dynamic stiffness instead of static stiffness to describe the spring’s ability to resist deformation under a dynamic load. The dynamic stiffness is the ratio of the response force amplitude to the displacement amplitude. The experiment was to apply different frequency sinusoidal displacement excitations to the PFA spring. And the excitation displacement was $u=u_{m}\sin\left(2\pi ft\right),$ $u_{m}$ was the excitation displacement amplitude, *f* was the excitation displacement frequency. After the spring vibration is stabilized, the corresponding force response amplitude $F_{m}$ can be obtained.

**Figure 5:**
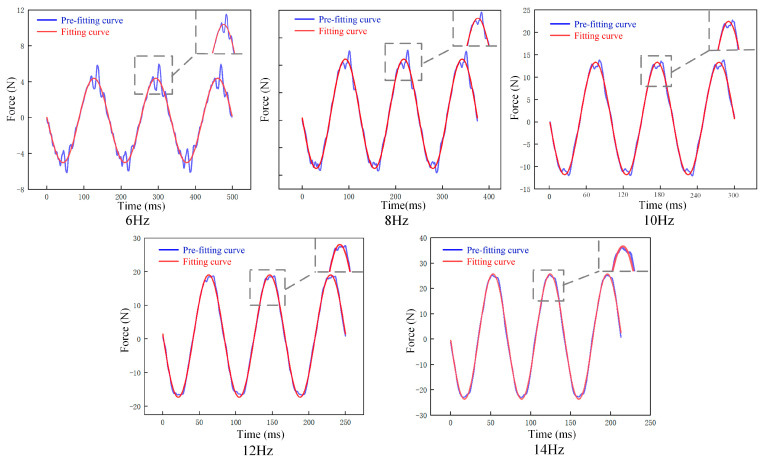
PFA spring force response and fitting force curve.

The force curves generated from 1000 cycles of excitation with frequencies ranging from 6 to 14 Hz are presented in Figure 5. As shown in Figure 5, the response force frequency of the spring was observed to be identical to the excitation frequency after 1000 cycles. The force response data collected at the maximum displacement position of the spring exhibited fluctuations. To obtain more accurate dynamic stiffness results, the collected force response experimental data were nonlinearly fitted using Formula (4), and the fitted curve is shown in Figure 5.

(4) $$F=F_{0}+F_{m}\times sin\left(2\pi ft-\phi \right)$$

The spring has a pre-compression amount, and the corresponding spring has an initial force response $F_{0}$. $F_{m}$ was the amplitude of the reaction force related to the external excitation frequency. Furthermore, f was the force response frequency, the same as the excitation displacement frequency. The phase of the response force lags behind the excitation displacement ϕ.

**Figure 6:**
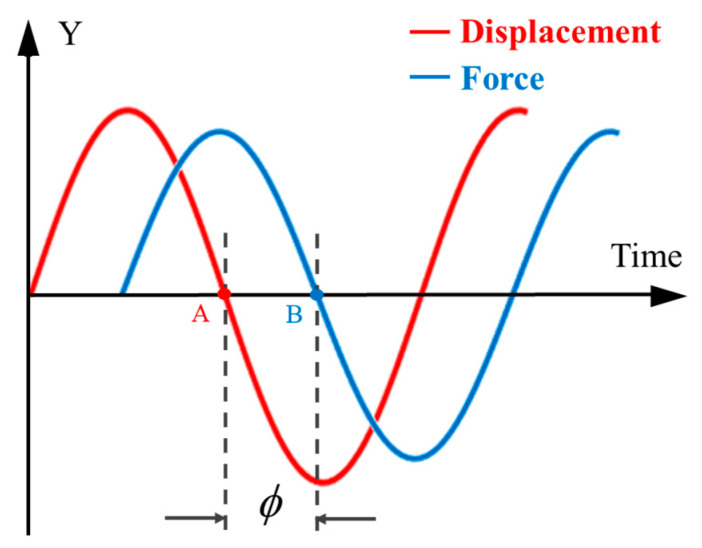
Phase difference diagram.

As shown in Figure 6, the fitting curve is in good agreement with the experimental curve. An accurate reaction force amplitude ($F_{m}$) can be obtained.

The dynamic stiffness can be written as follows:

(5) $$k_{d}=\frac{F_{m}}{u_{m}}$$

**Figure 7:**
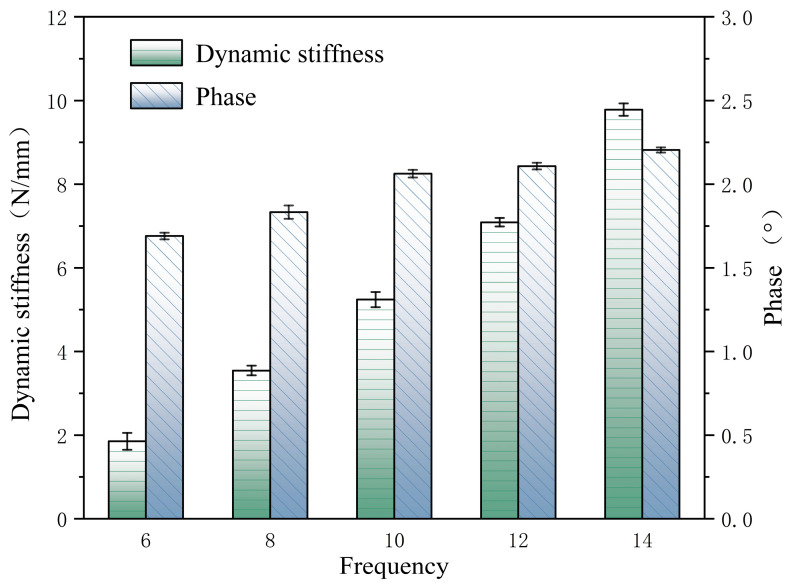
Dynamic stiffness under different cyclic frequencies.

Because the frequency of the force signal and the displacement signal is the same, the phase difference between the two remains constant. From Figure 6, the phase difference angle (ϕ) between the force signal and the displacement signal can be obtained. Figure 7 and Table 5 show the dynamic stiffness and the phase.

As shown in Table 5, the dynamic stiffness increases with the frequency. The small phase angles measured at different frequencies during testing indicate that the PFA spring has a small phase lag or phase difference and, thus, exhibits a higher sensitivity.

### 3.2. Effect of Frequency on Dissipation Coefficient

The dissipation coefficient of the check valve spring in a pump is a critical factor that can significantly influence the fluid flow in the valve. A suitable energy dissipation factor can ensure the system’s normal operation and optimal performance.

**Figure 8:**
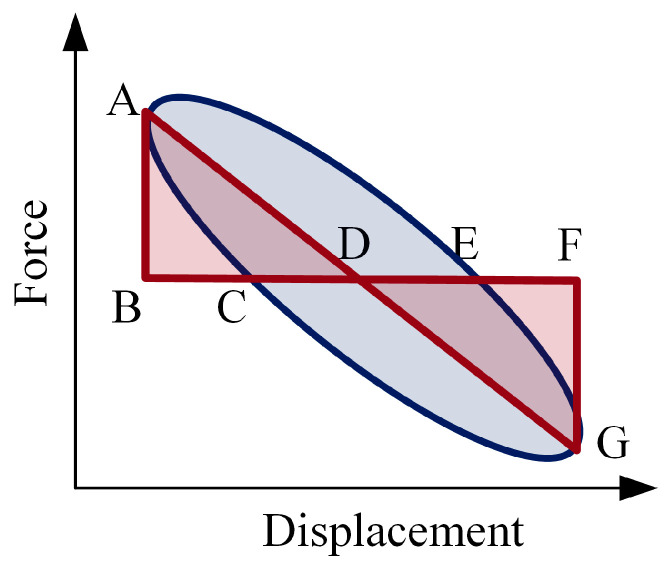
Diagram of the hysteresis curve.

The dissipation coefficient of a spring is usually analyzed based on specific hysteresis loops. The hysteretic loop curve refers to the displacement–force response curve during loading that does not coincide with the curve during unloading [27,28]. The loading curve and unloading curve constitute a closed loop. The phenomenon reflects the structure’s deformation characteristics, stiffness degradation, and energy consumption during repeated loading. The diagram of the hysteresis curve is shown in Figure 8. The dissipation coefficient is calculated based on the internal area of the hysteresis loop (*S*(*ACE* + *CEG*)) and the sum of the triangle area (*S*(*ABD* + *DFG*)). The hysteresis loop represents the energy dissipated in a cycle, and the sum of the triangles represents the elastic energy stored in a cycle. The dissipation coefficient can be written as follows [29]:

(6) $$E=\frac{S\left(ACE+CEG\right)}{S\left(ABD+DFG\right)}$$

**Figure 9:**
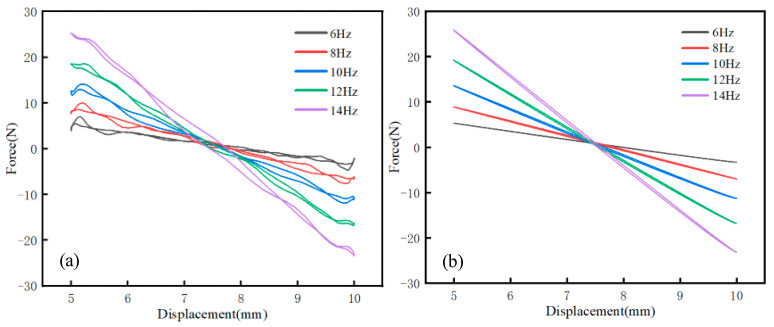
PFA spring force–displacement curve: (**a**) pre-fit curve, (**b**) fitting curve.

According to data collected from the experiment, the force–displacement curves of different excitation frequencies were drawn, as shown in Figure 9a. The PFA spring has noticeable hysteresis during repeated loading and unloading. The curve before fitting presents an irregular shape that differs from that mentioned in Section 2.2. Formula (6) could not be directly used to calculate the dissipation coefficient.

**Figure 10:**
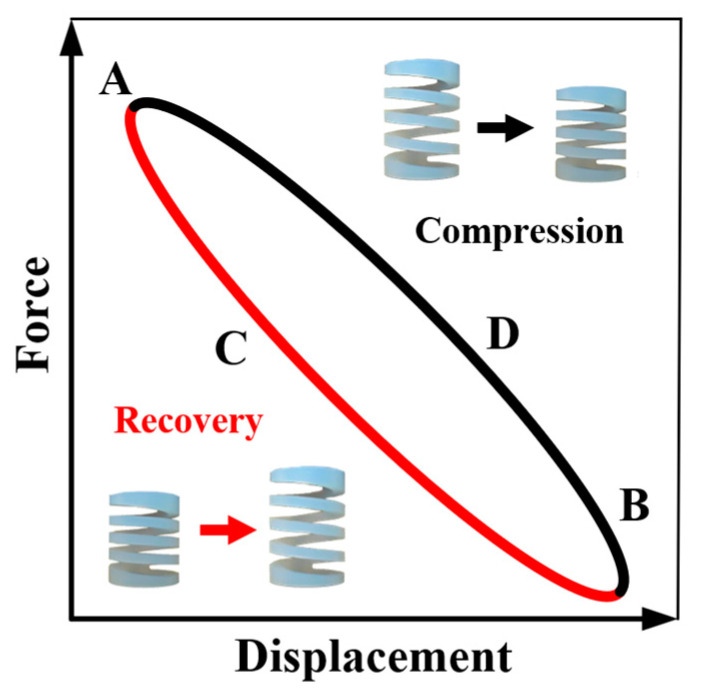
PFA spring force–displacement curve.

To calculate the energy dissipation factor, an orthogonal algorithm and sine fitting were applied to obtain a symmetrical fitted curve, as shown in Figure 9b. It was found that the fitting curve is symmetrical shape. The fitted curve can better represent the hysteresis law of the spring and facilitate the following calculation of the energy dissipation coefficient. According to Formula (6), the analysis software calculated the corresponding area of each frequency curve after fitting, and the energy dissipation coefficient was obtained and is recorded in Table 6. Under a cyclic compression load, the unloading stress–strain curve and the reloading stress–strain curve form a hysteresis curve ring, as shown in Figure 10. The line segment BDA is the PFA spring’s compression process, and the line segment ACB is the spring’s recovery process.

The results showed that the energy dissipation factor increased from 6 to 10 Hz and decreased from 10 to 14 Hz within the range of 6–14 Hz displacement excitation. The dissipation coefficient was the smallest at 6 Hz and reached the maximum at 10 Hz. In the range of 6 to 14 Hz displacement excitation, under 10 Hz displacement excitation, the energy consumed in one cycle of compression and recovery of spring load was the highest.

The energy dissipation of a PFA spring may be caused by the material damping temperature, the air resistance, and the geometry of the spring. In this experiment, the geometry of the spring was the same, and the springs were in a 320 K thermostat for the compression experiments. The excitation frequency will cause a change in the damping of the PFA material. The energy dissipation of the PFA spring is primarily due to material damping. During cyclic loading and deformation, some PFA molecules that make up the spring will rearrange, which requires energy consumption. Not all the energy is released during unloading, and some energy is converted into heat and lost to the surroundings. The frequency of excitation influences the behavior of the PFA molecules. When the frequency is shallow, the chain motion of the polymer can ultimately keep up with the change in the external force; the internal friction is minimal, and the polymer shows the high elasticity of the rubber. When the frequency is very high, the segment motion can keep up with the change in the external force; the internal friction is minimal, and the polymer appears rigid, showing the mechanical properties of the glassy state [30]. Only in the middle region, the segment motion cannot keep up with the change in the external force, and the internal friction will have a maximum value in a specific frequency range. The hysteresis phenomenon of the material in this region is pronounced. PFA spring should be avoided in the 10 Hz excitation frequency when choosing a working environment.

### 3.3. Effect of Frequency on Spring Dynamic Relaxation

With an increase in the cyclic excitation, the performance of the spring will change. The permanent free-height deformation of the spring is one of the ways of failure of the spring. Therefore, it is necessary to study the dynamic performance and free height of the PFA spring with the increase in cycle times. Let the free height of the spring be $H_{0}$, and the given free height be $H_{i};$ then, with a corresponding working load F and the relationship with the free height, the working height is

(7) $$F_{i}=k_{d}\left(H_{0}-H_{i}\right)$$

It can be seen from the above Equation (7) that when a PFA spring undergoes permanent deformation, such as a decrease in the free height $H_{0}$, the working load will also decrease for a given working stiffness value. The working load decreases when the dynamic spring stiffness decays, i.e., k decreases. Therefore, studying the variation in spring free height and dynamic stiffness with the increasing number of cycles at different excitation frequencies is necessary.

**Figure 11:**
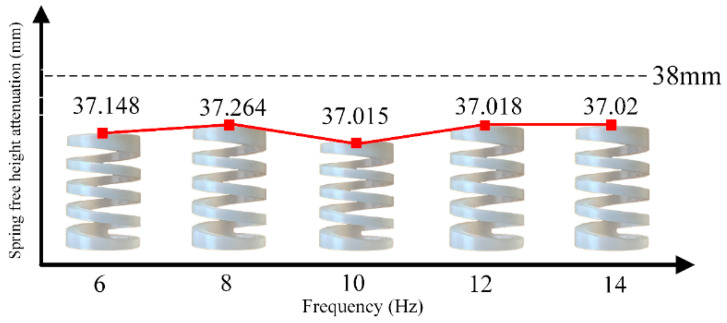
Spring free-height attenuation.

The PFA spring’s dynamic stiffness decay results are presented in Table 7, while the free-height variation tests are recorded in Figure 11. As explained in Section 3, numerical analysis software was utilized to calculate the spring’s energy decay rates at 10 thousand and 2 million loads, and the findings are documented in Table 8. These results offer significant insights into the PFA spring’s dynamic behavior and long-term performance characteristics.

The outcomes in Table 7 reveal that the dynamic stiffness of the PFA spring decays with an increasing number of cycles. The maximum decay in spring force is observed at 8 Hz, while the least decay is at 14 Hz. Figure 8 demonstrates that the free height of the PFA spring also diminishes as the number of cycles increases, with the most significant reduction observed at 10 Hz and the least at 6 Hz. Moreover, Table 8 shows that the spring’s energy dissipation coefficient is the highest for a 10 Hz excitation, consuming the most energy in one cycle and decaying the slowest. In contrast, a 6 Hz excitation exhibits the smallest spring ductility coefficient, consumes the least energy in one cycle, and decays the fastest.

These findings indicate that the structure and performance of the PFA spring alter as the number of cycles increases. The PFA spring’s free height decreases, its dynamic stiffness decreases, and its damping internal consumption decreases with increasing displacement excitation cycles. Notably, the most significant degradation in spring performance occurs at 10 Hz, so it is crucial to select a spring operating frequency that avoids this frequency range. Overall, by comprehending how the PFA spring responds to cyclic loading and deformation, it is possible to identify suitable operating conditions to ensure the optimal performance and longevity of the valve system.

## 4. Conclusions

In this study, the dynamic performance of the spring was investigated by conducting uniaxial cyclic mechanical tests at different frequencies and using indicators such as spring dynamic stiffness, energy dissipation coefficient, and performance attenuation rate. The following conclusion were drawn from the present study:

(1) In the range of experimental tests and operating frequencies, the dynamic stiffness of the PFA spring gradually increases as the excitation frequency increases.
(2) By analyzing the hysteretic curve of the PFA spring, the frequency is an essential factor affecting the energy dissipation coefficient of the PFA spring. The ductility coefficient of the PFA spring increases first and then decreases in the range of 6 to 14 Hz and is the smallest at 6 Hz and the largest at 10 Hz.
(3) Through the dynamic relaxation analysis of the PFA spring by the hysteresis curve, it is found that the frequency will affect the dynamic relaxation performance of the spring. The spring’s stiffness, free height, and ductility coefficient will decrease with the increase in the number of cyclic excitations. The PFA spring’s performance decays fastest under the excitation frequency of 10 Hz, and the work environment should avoid this frequency band.

In further studies, tensile spring tests should be performed to measure dynamic performance, and it is necessary to consider the effect of temperature on the spring.

## Figures and Tables

**Table 1:** Mechanical properties of PFA [25].

Properties	Values
Density (kg/m^−3^)	2150
Young’s Modulus (Pa)	5.516 × 10^8^
Poisson’s Ratio	0.46
Tensile Yield Strength (Pa)	9 × 10^6^
Compressive Yield Strength (Pa)	1.5 × 10^7^
Tensile Ultimate Strength (Pa)	3 × 10^7^

**Table 2:** Design parameters of spring structures.

Design Parameter (mm)	a	b	n	D	H
Value	4	3	3.5	25	38

**Table 3:** Static stiffness of PFA spring.

	1	2	3	Mean Value
Static stiffness (N/m)	119.45	121.26	121.74	120.82

**Table 4:** Compression parameters.

Test Parameters	Value
Excitation frequency (Hz)	6, 8, 10, 12, 14
Eexcitation magnitude (mm)	5

**Table 5:** Dynamic stiffness under different cyclic frequencies.

Frequency	6 Hz	8 Hz	10 Hz	12 Hz	14 Hz
Dynamic stiffness (N/mm)	9.789	3.545	5.238	7.088	9.780
Phase φ (°)	1.639	1.832	2.062	2.107	2.2043

**Table 6:** Spring dissipation coefficient.

Frequency	6 Hz	8 Hz	10 Hz	12 Hz	14 Hz
$E_{d}$	0.01308	0.01373	0.01935	0.01757	0.01746

**Table 7:** Maximum force attenuation.

Frequency	10 Thousand	2 Million	Decay Rate
6 Hz	5.27 N	5.21 N	1.14%
8 Hz	9.07 N	8.69 N	4.19%
10 Hz	13.56 N	13.29 N	2.01%
12 Hz	19.25 N	18.82 N	2.23%
14 Hz	25.96 N	25.90 N	0.23%

**Table 8:** Ductility coefficient attenuation.

Frequency	10 Thousand	2 Million	Decay Rate
6 Hz	0.01308	0.00757	42.1%
8 Hz	0.01373	0.009426	31.3%
10 Hz	0.01935	0.01514	21.8%
12 Hz	0.01757	0.01291	26.5%
14 Hz	0.01746	0.01276	26.9%

## Data Availability

Not applicable.
